# Perceiving, interpreting and responding to changes in hand function among older adults with mild cognitive impairment: a common-sense model-informed qualitative study

**DOI:** 10.3389/fpsyt.2026.1873688

**Published:** 2026-09-21

**Authors:** Jingjing Yan, Tingting Yin, Yan Wang, Zishuo Mi, Haiyan Yin, Yulei Song, Yamei Bai

**Affiliations:** College of Nursing, Nanjing University of Chinese Medicine, Nanjing, China

**Keywords:** common-sense model, functional vulnerability, hand function, illness representation, mild cognitive impairment, older adults, qualitative study

## Abstract

**Background:**

Older adults with mild cognitive impairment (MCI) may exhibit selective differences in hand function, including complex manual dexterity, movement speed, rhythm, and performance variability, even while basic independence remains largely preserved. However, existing research has relied predominantly on objective measures, and little is known about how older adults notice, interpret, and respond to these changes in everyday life. Guided by the Common-Sense Model of Self-Regulation (CSM), this study explored the subjective experiences and everyday meanings of changes in hand function among community-dwelling older adults with MCI.

**Methods:**

A descriptive qualitative study was conducted. Nineteen community-dwelling older adults with MCI were recruited from three communities in Nanjing, China. Semi-structured interviews were conducted and analyzed using directed content analysis informed by the CSM. The study followed the COREQ checklist.

**Results:**

Seven themes were identified and mapped to the relevant CSM domains: (1) identity—changes becoming visible through disruption in familiar activities; (2) timeline—gradual changes absorbed into everyday adaptation; (3) cause—aging as a normalizing explanation for changes in hand function; (4) consequences—consequences emerging through functional participation and self-identity; (5) control and coping—self-adjustment, support seeking, and limited pathways for action; (6) coherence—hand–cognition links interpreted through experiential knowledge; and (7) emotional representations—normalization, loss, and determination. Together, these themes described variation in how participants recognized changes in hand function, understood their causes and consequences, related them to cognition, and responded emotionally and behaviorally.

**Discussion:**

Everyday activities both made subtle changes in hand function visible and gave them practical meaning. Aging provided a familiar causal explanation, but this explanation was not always integrated into a coherent understanding of the nature, possible cognitive relevance, and controllability of these changes. Accounts of concrete coping more often linked valued activities with feasible strategies and available support. These findings suggest that noticing change, making sense of it, and knowing how to respond are interrelated yet distinguishable components of self-regulation and may inform activity- and goal-oriented approaches to assessment and care planning.

## 1 Introduction

Mild cognitive impairment (MCI) is characterized by objective decline in one or more cognitive domains beyond that expected from normal aging, while independence in everyday activities remains largely preserved and the criteria for dementia are not met (1, 2). In China, approximately 15.5% of adults aged 60 years or older have MCI, making it an important public health concern (3). The heterogeneous cognitive profile of MCI may involve memory, attention, executive function, processing speed, language, and visuospatial abilities, and decline across these domains may affect performance in complex everyday activities (1). Executive function illustrates this relationship: lower executive performance in cognitively normal older adults has been identified as one of several predictors of subsequent progression to MCI, whereas executive dysfunction in people with MCI has been independently associated with poorer instrumental activities of daily living (4, 5). Such difficulties may initially manifest as slower, less accurate, or less efficient performance in cognitively demanding activities, including medication management, financial tasks, appointment keeping, and technology use, rather than as a complete inability to perform them (6, 7). Even at this subtle stage, functional difficulties may affect continued independent living and increase the need for family support and caregiving (8). Although the clinical course is heterogeneous and progression to dementia is not inevitable, MCI represents an important stage at which to examine emerging functional vulnerability before substantial dependence develops (9).

Hand function is fundamental to independent living because eating, dressing, preparing food, taking medication, and many other everyday activities require effective use of the hands. It is multidimensional, encompassing grip and pinch strength, tactile sensation, fine force control, manual dexterity, and movement speed; age-related musculoskeletal, sensory, and neural changes can reduce the precision, speed, and efficiency of hand use even among otherwise independent older adults (10, 11). Nevertheless, hand problems among community-dwelling older adults may remain unassessed or untreated until later stages, while routine clinical assessment commonly emphasizes maximum grip strength rather than broader sensorimotor components (12, 13). In a survey of 595 community-dwelling older adults, 85% considered hand function important, but only 43% were concerned about losing it; one-third had considered seeking care for a hand-related limitation but did not, most commonly because they did not consider their function poor enough to warrant care (14). Thus, changes in hand function can have meaningful consequences in later life but may remain insufficiently recognized or addressed. Evidence from older adults generally, however, cannot establish whether changes occurring in MCI reflect age-related decline alone or additional vulnerability associated with cognitive impairment.

Evidence from MCI populations suggests that hand-function differences may be selective rather than generalized, particularly in complex dexterity, movement speed, rhythm, and performance variability. A broader cognitive–motor basis is suggested by associations between hand function and cognitive performance, particularly executive function, among older people with MCI or dementia (15). Compared with cognitively healthy older adults, those with MCI have demonstrated fewer finger taps, longer inter-tap intervals, greater performance variability, and altered tapping rhythm (16, 17). Among functionally independent older adults, slower performance on a snap-fastener task was associated with MCI and impairments in memory, attention, executive function, and processing speed, whereas grip strength was not significantly associated with cognitive impairment (18). Findings also vary by task and MCI subtype: slower fine motor dexterity has been reported in multidomain but not single-domain amnestic MCI, while psychomotor speed may be affected even when grip strength and spiral tracing remain relatively preserved (19, 20). Taken together, these findings suggest that task- and domain-dependent hand-function vulnerability may become more apparent as cognitive–motor demands increase and may be present while basic independence remains largely preserved, underscoring the importance of examining how such subtle changes are experienced in everyday life.

Existing research has primarily quantified hand performance and examined its association with cognitive function, with less attention to how older adults with MCI notice, interpret, and respond to changes in everyday life. Awareness and appraisal of functional changes may vary across individuals and contexts and may be reduced in some cases (21, 22). It therefore remains unclear when changes in hand function become noticeable, how older adults explain them, whether they relate them to aging or cognitive health, and how they respond. Qualitative inquiry is needed to examine these experiences from the perspectives of older adults with MCI themselves.

Leventhal and colleagues’ Common-Sense Model of Self-Regulation (CSM) provides the theoretical framework for this inquiry (23). Developed within health psychology, the CSM is a self-regulation framework that explains how individuals form representations of health-related cues and how these representations inform emotional responses, coping, and subsequent appraisal. These beliefs are shaped by bodily sensations, previous experience, sociocultural context, and external information and are organized around identity, timeline, cause, consequences, control, and coherence. The model has been applied to research on symptom perception, illness representations, self-management, and health behavior (24–26). The CSM is particularly suited to the present study because it links representations of subtle functional cues with emotional and coping responses, allowing changes in hand function to be examined both as performance difficulties and as experiences interpreted in relation to aging, cognitive health, and continued independence. Guided by the CSM, this study examined how community-dwelling older adults with MCI: (a) identified and described changes in hand function; (b) understood their timeline, causes, consequences, and controllability; (c) interpreted the relationship between hand function and cognitive function; and (d) experienced emotional responses and adopted everyday coping strategies. The study aimed to inform more context-sensitive approaches to functional assessment, communication, and support.

## 2 Methods

### 2.1 Study design

This study used a descriptive qualitative design to explore how older adults with MCI perceived, interpreted and responded to changes in hand function in everyday life. The CSM informed the development of the interview guide and provided the initial analytic orientation. The study was reported in accordance with the Consolidated Criteria for Reporting Qualitative Research (COREQ) (27), and the completed checklist is provided as **Supplementary File 1**.

### 2.2 Participants and recruitment

Purposive sampling was used to recruit community-dwelling older adults with MCI from three communities in Nanjing, China, between November 2025 and January 2026. Participants were recruited to achieve variation in gender, age and educational background. Recruitment posters were displayed in community activity centers and community health service stations, and community staff assisted with the preliminary identification of potential participants. The research team then contacted interested individuals, explained the study purpose and procedures, screened them according to the eligibility criteria, confirmed their willingness to participate and arranged the interview time and location.

### 2.3 Inclusion and exclusion criteria

The inclusion criteria for community-dwelling older adults with MCI were as follows: (1) age ≥60 years; (2) MCI operationally defined according to the following criteria: (a) subjective cognitive decline reported by the participant and a primary informant; (b) objective cognitive impairment on screening, assessed using the Montreal Cognitive Assessment (MoCA) and defined as a total score of <26, with one additional point added for individuals with ≤12 years of education in accordance with the standard scoring guidelines (28); (c) preserved basic functional independence, assessed using the Activities of Daily Living Scale (ADL), with a score of <26 indicating no significant functional impairment; and (d) absence of a prior clinical diagnosis of dementia (29); (3) clear consciousness and adequate communication ability; and (4) provision of written informed consent and voluntary participation. The exclusion criteria were as follows: (1) a diagnosis of a severe psychiatric disorder; (2) clinically significant depressive or anxiety symptoms, defined as a score of ≥8 on the 15-item Geriatric Depression Scale (GDS-15) or ≥14 on the Hamilton Anxiety Rating Scale (HAMA), respectively (30, 31); (3) severe hearing or visual impairment; and (4) hand dysfunction attributable to identifiable conditions such as rheumatoid arthritis, hand trauma, or neurological disorders.

Sampling and analysis proceeded concurrently. Following the analysis of interview 17, two additional interviews were conducted. Neither interview 18 nor interview 19 generated new themes or information that meaningfully extended the existing categories; recruitment therefore ceased after the 19th interview.

### 2.4 Researcher characteristics and reflexivity

Interviews were conducted by the first author, Jingjing Yan (J.Y.), a female postgraduate nursing student who had received training in qualitative research and was supervised by senior researchers experienced in aging research and qualitative methods. The interviewer had not met the participants before recruitment. Before each interview, she introduced her research role, explained the purpose of the study, and established rapport with the participant.

The interview guide was developed collaboratively by J.Y. and two nursing researchers with relevant methodological and subject-area expertise: Tingting Yin (T.Y.), a doctoral candidate in nursing with research experience in gerontological nursing, community nursing, MCI, and qualitative methods; and Yulei Song (Y.S.), PhD, Associate Professor, with interdisciplinary research experience in MCI, psychology, and public health. These three researchers reviewed the guide for its alignment with the study aims and the CSM framework, the relevance and clarity of its content, and its suitability for community-dwelling older adults with MCI. The remaining authors (Y.W., Z.M., H.Y., and Y.B.) contributed to discussions of emerging findings and to the review of coding decisions and theme development; the primary coding responsibilities of J.Y., T.Y., and Y.S. are described in Section 2.6.

To enhance reflexivity, J.Y. recorded field notes after each interview, including contextual observations and preliminary reflections. During data collection and analysis, the research team held regular discussions to examine assumptions, compare interpretations, and reduce the influence of individual preconceptions on the analysis.

### 2.5 Data collection

A semi-structured interview guide was developed based on the study aims, literature review and the CSM framework. The guide included open-ended questions about participants’ recognition and description of changes in hand function, perceived timeline, possible causes, consequences, controllability, relationship between hand function and cognition, emotional responses and coping strategies. The interview guide is presented in **Table 1**.

**Table 1:** Semi-structured interview guide informed by the common-sense model of self-regulation.

Interview component	Questions
Opening	To begin, could you tell me about how you usually use your hands in your everyday life?
Identity	Can you tell me about any changes you have noticed in your hands in your daily life? How would you describe what your hands feel or function like now?
Timeline	Can you tell me when you first noticed these changes? How have these changes developed over time?
Cause	What do you think caused these changes?
Consequences	How do these changes affect your daily life?
Control	What do you usually do to cope with these changes?
Coherence	Do you think there is any relationship between changes in hand function and changes in memory or cognition?
Emotional representations	How do you feel when you notice or think about these changes?
Closing	Is there anything else about your hands or hand function in everyday life that you would like to add?

All interviews were conducted one-to-one and face-to-face in Mandarin Chinese. Interviews took place in quiet and relatively private community settings, or in familiar locations preferred by participants when appropriate. No family members or other non-participants were present during the interviews. With participants’ permission, all interviews were audio-recorded. Field notes were taken to document the interview context, non-verbal behaviors and the interviewer’s observations. Interviews lasted approximately 31 minutes on average (SD = 13.6; range, 21–77 minutes).

Audio recordings were transcribed verbatim in Chinese within 24 hours of each interview and checked against the recordings and field notes for accuracy. Data analysis was conducted using the original Chinese transcripts. Representative quotations used in the manuscript were translated into English by the first author and checked by a bilingual researcher to ensure semantic accuracy. Any discrepancies in translation were discussed within the research team. Translated quotations were not returned to participants because the analysis was conducted on the original Chinese data.

### 2.6 Data analysis

Directed content analysis was selected because the CSM provided an existing conceptual framework closely aligned with the study questions and could guide the initial interpretation of participants’ accounts (32). The CSM domains guided the initial analysis, while transcripts were coded line by line and the coding framework was refined iteratively to accommodate new or more specific meanings emerging from participants’ accounts.

J.Y. and T.Y. independently read and coded the transcripts from the first three interviews line by line. They compared their codes, discussed differences, and developed a preliminary coding framework. J.Y. then applied this framework to the subsequent transcripts, while T.Y. reviewed the coding decisions and proposed refinements. The framework was refined iteratively as new meanings emerged. Codes were compared across transcripts and grouped into subthemes reflecting recurring patterns in participants’ experiences. The subthemes were further abstracted into broader themes describing how older adults with MCI perceived, interpreted, and responded to changes in hand function.

Differences in coding and theme development were discussed by J.Y. and T.Y. When an issue remained unresolved, Y.S. joined the discussion, and the final decision was reached collectively. Regular team meetings were held to review coding decisions, refine themes, and ensure that the analysis remained grounded in participants’ accounts while being informed by the CSM. NVivo 15 (Lumivero, Denver, CO, USA) was used to support data management and coding (33).

### 2.7 Trustworthiness

Several strategies were used to enhance trustworthiness. First, purposive sampling was used to include participants with different demographic characteristics. Second, data collection and analysis were conducted concurrently, allowing emerging issues to inform subsequent interviews. Third, the credibility of the analysis was supported by independent line-by-line coding of the initial interviews by J.Y. and T.Y., review of the subsequent coding by T.Y., consensus discussions, and the involvement of Y.S. when disagreements remained unresolved. Finally, an audit trail was maintained, including audio recordings, transcripts, coding records, analytic memos, and theme-development documents.

### 2.8 Ethical considerations

This study was approved by the ethics committee of the authors’ institution (Approval No. 2025NZY-13-01; approved on 14 May 2025). All participants provided written informed consent before participation. Participants were informed that participation was voluntary and that they could withdraw at any time without consequence. Interview data were anonymized, and participants were identified by code numbers. Audio recordings, transcripts and coding files were stored securely by the first author on a password-protected computer, with access restricted to the research team.

## 3 Results

Nineteen eligible older adults with MCI completed the interviews. No participants withdrew after providing informed consent. Participants ranged in age from 68 to 82 years and varied in gender, educational background, marital status, living arrangement, monthly disposable income and pre-retirement occupational work type. Detailed participant characteristics are presented in **Table 2**. Detailed individual-level sociodemographic information is provided in **Supplementary Table 1**.

**Table 2:** Sociodemographic and clinical characteristics of participants (N = 19).

Characteristic	Mean (SD) or n (%)	Range
Age (years)	73.3 (4.3)	68–82
60–69	3 (15.8)	
70–79	14 (73.7)	
≥80	2 (10.5)	
Gender		
Male	7 (36.8)	
Female	12 (63.2)	
Years of education	12.1 (2.9)	6–16
MoCA score	23.0 (1.9)	18–25
Marital status		
Married	14 (73.7)	
Widowed	5 (26.3)	
Living arrangement		
Living alone	5 (26.3)	
Living with spouse	8 (42.1)	
Living with children	3 (15.8)	
Living with spouse and children	3 (15.8)	
Monthly disposable income, CNY/month		
<1,000	1 (5.3)	
1,000–2,000	5 (26.3)	
2,001–3,000	2 (10.5)	
3,001–4,000	4 (21.1)	
4,001–5,000	1 (5.3)	
>5,000	6 (31.6)	
Pre-retirement occupational work type		
Mental work	10 (52.6)	
Manual work	4 (21.1)	
Mixed mental and manual work	5 (26.3)	

Values are presented as mean (SD) and range for continuous variables and as n (%) for categorical variables. Monthly disposable income was collected using predefined categories and is therefore reported as n (%). CNY, Chinese yuan; MoCA, Montreal Cognitive Assessment.

Analysis of the interview data generated seven themes that described how older adults with mild cognitive impairment perceived, interpreted and managed changes in hand function in everyday life. Across themes, participants’ accounts showed that changes in hand function were usually noticed through disruptions in familiar daily activities rather than through abstract judgments about bodily ability. These changes were interpreted in different ways and were managed in ways that prioritized maintaining independence and continuity in daily life. **Table 3** provides an overview of the themes and subthemes identified in the analysis.

**Table 3:** CSM domains, themes and subthemes for changes in hand function in older adults with MCI.

CSM domain	Theme	Subthemes
Identity	Theme 1. Changes becoming visible through disruption in familiar activities	• Clearly recognized changes • No noticeable change • Uncertain awareness
Timeline	Theme 2. Gradual changes absorbed into everyday adaptation	• Gradual and imperceptible change • Anticipating future decline
Cause	Theme 3. Aging as a normalizing explanation for changes in hand function	• Aging and bodily decline • Illness and health conditions • Habits, emotions and environment
Consequences	Theme 4. Consequences emerging through functional participation and self-identity	• Reduced everyday efficiency • Avoidance and withdrawal • Threats to self-identity • Limited perceived impact
Control	Theme 5. Coping responses shaped by actionability and everyday priorities	• Self-adjustment and function maintenance • Seeking support • Limited action pathways
Coherence	Theme 6. Hand–cognition links interpreted through experiential knowledge	• Intuitive connections • Separate domains
Emotional representations	Theme 7. Normalization, loss and determination	• Calm acceptance and resignation • Frustration and loss • Hope and determination

Themes were developed from participants’ accounts and mapped to CSM domains.

### 3.1 Theme 1. Changes becoming visible through disruption in familiar activities (Identity)

Participants described a range of changes in hand function in everyday life (**Table 4**), primarily identified through disruptions in everyday activities. However, recognition of these changes varied across participants. Reported changes were grouped into four broadly defined aspects for clarity.

**Table 4:** Changes in hand function reported in everyday activities by older adults with MCI.

Aspect of change in hand function	Illustrative reported experiences in everyday activities
Hand strength and force-related function	Reduced grip or wrist strength; difficulty carrying, lifting, wringing towels, opening bottle caps or holding objects; objects slipping from the hand.
Manual dexterity, movement fluency and task performance	Reduced finger dexterity; slower or less smooth hand movements; difficulty picking up small objects, buttoning, sewing, peeling beans, turning pages, writing or completing detailed manual tasks; occasional difficulty coordinating intended actions with hand movements.
Hand stability and motor control	Trembling or shaking during writing, cutting vegetables, cooking, holding cups or using a phone; feeling that hand movements were less steady or less controlled.
Sensory perception and bodily sensations of the hand	Reduced tactile sensitivity, numbness, altered temperature perception, stiffness, dryness or roughness affecting hand use.

Categories summarize reported experiences and are broad descriptive categories, not a clinical classification. Examples are illustrative.

#### 3.1.1 Subtheme A: clearly recognized changes

Some participants clearly recognized changes in hand function, typically through disruptions in everyday activities. They described difficulties in activities requiring hand strength, such as wringing out towels and opening bottle caps; fine dexterity, such as picking up a single strand of hair; and timely hand movements, such as keeping pace while counting or doing housework. Their accounts were expressed in definite, functional terms and reflected noticeable differences from previous abilities.

*‘My grip strength is not what it used to be. I used to wring towels really dry, but now I just don’t have that strength anymore. Sometimes I can’t even twist open a bottle cap.’ (N17, female, 72 years)*

*‘When I comb my hair in the morning, sometimes a strand falls onto the windowsill and I can’t pick it up. When I was younger, I could pick up a single hair easily, but now my fingers just don’t feel as nimble as before.’ (N3, female, 73 years)*

*‘When I want to count quickly or do some calculations, my mind keeps up, but my hands don’t. It’s the same when I’m doing housework.’ (N15, male, 71 years)*

#### 3.1.2 Subtheme B: no noticeable change

In contrast, some participants did not perceive any obvious change in hand function and described their abilities as largely unchanged.

*‘I still do things like picking vegetables and shelling edamame. My hands are still quick and nimble enough.’ (N7, male, 68 years)*

*‘My hands don’t tremble. No matter what I’m doing, they don’t shake.’ (N16, female, 77 years)*

#### 3.1.3 Subtheme C: uncertain awareness

Some participants did not make a clear judgment about whether change had occurred. Rather than confidently reporting either change or stability, they expressed hesitation and uncertainty, especially when prompted to reflect on specific aspects of hand function.

*‘I don’t remember any changes … I haven’t paid attention to that. What kind of change? Maybe there is one? … I really haven’t noticed, so I don’t know.’ (N4, female, 82 years)*

Some participants offered tentative, situational explanations for this uncertainty, including limited involvement in housework and consequently paying less attention to their hand function.

*‘Maybe it’s really because I don’t do much housework, so I haven’t paid much attention to changes in my hands.’ (N11, male, 82 years)*

### 3.2 Theme 2. Gradual changes absorbed into everyday adaptation (Timeline)

Participants commonly described changes in hand function as gradual, subtle and ongoing, rather than sudden or clearly identifiable.

#### 3.2.1 Subtheme A: gradual and imperceptible change

Most participants experienced change as slow and almost imperceptible, without a clear starting point.

*‘It declined little by little. Gradually I found that I had less strength than before, and my hand flexibility was also not as good as it used to be.’ (N10, male, 70 years)*

*‘It changed very gradually, so at first I did not pay much attention to it.’ (N17, female, 72 years)*

#### 3.2.2 Subtheme B: anticipating future decline

Some participants also anticipated further deterioration over time.

*‘In another two years, it definitely won’t be okay. It’s the kind of thing that gets worse year by year.’ (N3, female, 73 years)*

*‘The changes in my hands have hardly affected my daily life. But maybe in a few more years, I won’t be able to manage so well.’ (N1, female, 75 years)*

### 3.3 Theme 3. Aging as a normalizing explanation for changes in hand function (Cause)

Participants drew on multiple explanations to make sense of changes in hand function. Although aging was the most common attribution, other explanations included illness, bodily conditions, personal habits and environmental changes.

#### 3.3.1 Subtheme A: aging and bodily decline

Aging was the most common explanation for changes in hand function and was used by participants regardless of whether they had clearly recognized such changes. Participants associated this explanation with specific age-related bodily changes, particularly reduced hand strength and declining vision, which affected activities such as opening bottle caps and threading a needle.

*‘Now that I am older, my eyesight has worsened and I cannot see clearly. My hands do not work as well when I try to thread a needle.’ (N3, female, 73 years)*

*‘I think it is due to aging. Now that I am older, the strength in my hands is clearly not what it used to be. I used to open bottle caps easily, but now it takes a lot of effort. I think my strength has declined as I have aged.’ (N15, male, 71 years)*

#### 3.3.2 Subtheme B: illness and health conditions

Some participants linked changes in hand function to chronic conditions or other bodily impairments.

*‘Sometimes my fingers don’t feel very nimble, but that’s mainly because my eyesight isn’t very good. If I’m wearing the right glasses, I can do things quite smoothly; if not, I’m more likely to make mistakes, especially with fine tasks like knitting.’ (N10, male, 70 years)*

*‘My lumbar spine and cervical spine are not in very good shape, so I can’t carry heavy things, and my hands also feel weak.’ (N2, female, 68 years)*

*‘The changes in my hand function may be related to my diabetes.’ (N5, male, 72 years)*

#### 3.3.3 Subtheme C: habits, emotions and environment

Participants also drew on personal habits, psychological experiences and environmental changes to make sense of hand function.

*‘To be honest, I was never good at delicate work even when I was young.Well, that’s a common thing with men — we’re just not that meticulous when it comes to doing things.’ (N6, male, 70 years)*

*‘Now everything is bought. It’s caused by the social environment. We don’t do many things anymore, except cooking something to eat.’ (N1, female, 75 years)*

In one case, hand tremor was attributed to psychological distress after a major life event:

*‘It began after my husband’s accident … my hands started shaking, and I didn’t know why at first. Later the doctor told me it might be related to psychological factors.’ (N19, female, 76 years)*

### 3.4 Theme 4. Consequences emerging through functional participation and self-identity (Consequences)

Participants did not describe the consequences of changes in hand function simply as losses in isolated hand abilities. Rather, these consequences became apparent through changes in functional participation, activity engagement and their sense of competence or self-identity.

#### 3.4.1 Subtheme A: reduced everyday efficiency

For participants who recognized changes, these were most commonly experienced as reduced efficiency in everyday activities.

*‘Something that used to take half an hour now can’t be finished in half an hour.’ (N1, female, 75 years)*

#### 3.4.2 Subtheme B: avoidance and withdrawal

Some participants avoided activities that had become more difficult to manage or withdrew from activities that required sustained cognitive and fine manual coordination.

*‘Things are not as easy for me as they used to be. I used to carry groceries home from the market, but now I do not want to go shopping unless I take a shopping trolley.’ (N1, female, 75 years)*

*‘I used to knit frequently when I was younger, but I no longer knit now. Making patterned designs requires working out how to alternate between different stitches. These days, for one thing, I do not feel like thinking things through as much; for another, my fingers have become slower and are not as flexible as before.’ (N3, female, 73 years)*

#### 3.4.3 Subtheme C: threats to self-identity

For some, changes in hand function were experienced not only as functional decline but also as a challenge to their previous sense of self.

*‘When I realized my grip was no longer as good as before, I felt that I had become old.’ (N17, female, 72 years)*

*‘When I look at these characters and realize I wrote them, it feels as if they’re no longer mine.’ (N1, female, 75 years)*

#### 3.4.4 Subtheme D: limited perceived impact

At the same time, a considerable number of participants perceived the impact of changes in hand function as limited. Although they were aware of differences in hand function, these changes were not experienced as disruptive to everyday life.

*‘The changes in my hands basically don’t affect my life. They only shake a little when I write. Apart from that, it doesn’t affect housework.’ (N12, female, 75 years)*

*‘I don’t need to do delicate work … I only do rough work, so there’s no real pressure.’ (N8, female, 71 years)*

### 3.5 Theme 5. Coping responses shaped by actionability and everyday priorities (Control)

Participants’ responses to changes in hand function were shaped not only by the presence of difficulty, but also by whether they could identify feasible ways to respond and by which abilities they considered most important for maintaining everyday independence.

#### 3.5.1 Subtheme A: self-adjustment and function maintenance

Many participants described practical adjustments to manage current difficulties in daily life. These included using assistive tools, changing the way tasks were performed, and selectively exercising the abilities they considered most important.

*‘Now I use a cart when shopping so I don’t have to carry things by hand.’ (N1, female, 75 years)*

*‘These days, if clothes are fiddly to button up, I’d rather not wear them. It feels like too much hassle. When I buy clothes, I prefer ones with zippers — they’re much easier and more convenient.’ (N14, female, 68 years)*

Some participants also made self-initiated efforts to maintain hand function.

*‘I use a spring hand gripper to train my hands.’ (N13, male, 79 years)*

*‘Here in the community where I live now, I take part in Baduanjin, Tai Chi sword, and Tai Chi … I think they’re good because they help improve the flexibility of my hands.’ (N1, female, 75 years)*

Participants did not try to exercise all aspects of hand function equally, but tended to prioritise abilities they considered most relevant to everyday independence.

*‘What I most want to work on is the ability to write and do housework. Of these, flexibility is the most important. Touch is a minor thing, and strength only needs to be enough for daily needs. If my hands are no longer flexible, daily life becomes inconvenient.’ (N11, male, 82 years)*

#### 3.5.2 Subtheme B: seeking support

Many participants also sought help from others when tasks became difficult. Support was commonly provided by family members, but could also come from people outside the home.

*‘Nowadays, if there’s something I can’t manage easily, I ask my husband to help me.’ (N8, female, 71 years)*

*‘If I buy something with a lid, I get the staff in the supermarket to open it before I take it home.’ (N3, female, 73 years)*

Participants generally viewed such help as practical and acceptable.

*‘It’s normal to ask others for help when you can’t manage.’ (N16, female, 77 years)*

#### 3.5.3 Subtheme C: limited action pathways

A minority of participants described limited coping responses when faced with hand-related difficulties. Rather than actively seeking strategies to improve hand function, they tended to continue as usual, leave difficult tasks unfinished, or express uncertainty about what could be done.

*‘What else could I do? I didn’t think about making any changes. I just wrote as usual and didn’t really bother about it.’ (N8, female, 71 years)*

*‘I haven’t really thought about training my hands. I don’t know what else I could do, or what I could do to improve it.’ (N18, female, 72 years)*

### 3.6 Theme 6. hand–cognition links interpreted through experiential knowledge (Coherence)

Participants varied in how they understood the relationship between hand function and cognition. Some regarded the relationship as meaningful, whereas others saw the two as separate.

#### 3.6.1 Subtheme A: intuitive connections

Some participants felt that hand function and cognition were connected, even if they could not always explain this clearly. Their understanding was shaped by everyday experience, cultural beliefs and health-related information.

*‘Exercising the hands should be able to exercise the brain, because the brain is the command center, and hand activity can stimulate the brain.’ (N15, male, 71 years)*

*‘There’s an old saying that ‘clever hands reflect a clever mind.’ If the hands are skillful, the brain is agile; if the hands are not flexible, the brain will also be affected.’ (N17, female, 72 years)*

*‘I saw on my phone that doing finger exercises can train the brain, because moving the fingers also stimulates the brain. So I do think there is a connection, although I can’t explain exactly how.’ (N13, male, 79 years)*

#### 3.6.2 Subtheme B: separate domains

Other participants regarded hand function and cognition as separate processes.

*‘One is the brain, the other is strength. They are two different things.’ (N3, female, 73 years)*

*‘I don’t think hand condition has anything to do with memory. These are two different things. Intelligence is about the brain nerves, while the hand is about the limbs.’ (N7, male, 68 years)*

### 3.7 Theme 7. Normalization, loss and determination (Emotional representations)

Participants described a range of emotional responses to hand-related change, including acceptance, resignation, frustration, loss and determination.

#### 3.7.1 Subtheme A: calm acceptance and resignation

Many participants described changes in hand function as a normal part of aging. For some, this was expressed relatively calmly; for others, it was accompanied by helplessness or quiet resignation.

*‘It doesn’t really affect my mood. I just don’t care – I think as you get older, things gradually become like this. It’s the natural way.’ (N12, female, 75 years)*

*‘It can’t be compared with when I was younger. After all, I’m older now — this is just how it is. There’s nothing you can do about it. You just have to face reality and carry on with life.’ (N7, male, 68 years)*

#### 3.7.2 Subtheme B: frustration and loss

Some participants described broader negative emotional responses, particularly when they could no longer perform familiar tasks as before.

*‘Things I used to be especially good at, I can no longer do well now. I feel a little disappointed, but I don’t know how to improve it.’ (N5, male, 72 years)*

#### 3.7.3 Subtheme C: hope and determination

Alongside acceptance and loss, some participants expressed hope, positivity and persistence.

*‘This decline has not really affected my daily life very much. People are like this when they get old. The most important thing is to keep a good mood — when your mindset is good, everything is easier to deal with.’ (N15, male, 71 years)*

*‘I used to be able to carry more than 100 jin, but now I can’t. Sometimes I still feel unwilling to accept it, so I want to try again and see whether I can still lift it.’ (N10, male, 70 years)*

## 4 Discussion

This study used the CSM to explore how community-dwelling older adults with MCI perceived, interpreted, and responded to changes in hand function in everyday life. Three interrelated insights emerged. First, everyday activities served both as contexts in which subtle changes in hand function became recognizable and as reference points for judging their practical significance. Second, aging provided a familiar and readily understandable causal explanation, yet these changes occupied an ambiguous interpretive space between aging and cognitive health. Third, variation in coping was most evident in whether participants could connect valued activities or abilities with feasible strategies and available support; the responses described mainly centered on maintaining or modifying familiar activities and reducing engagement in them. Taken together, these findings provide new insight into how subtle changes in hand function acquire varying degrees of visibility, interpretive coherence, and actionability in the context of MCI. They also indicate that noticing a change, making sense of it, and knowing how to respond are interrelated yet distinguishable components of self-regulation.

### 4.1 The dual role of everyday activities in recognizing hand-function change and evaluating its practical significance

In this study, everyday activities were not only settings in which changes in hand function had consequences but also experiential reference points through which subtle changes could be recognized. Familiar activities enabled participants to compare their current performance with their previous abilities, thereby making gradual and indistinct bodily changes recognizable as specific functional differences. Conversely, limited engagement in relevant activities or gradual habituation to changes through ongoing adjustment may have reduced opportunities for such comparisons. From a CSM perspective, bodily sensations need to acquire recognizable features and meaning before they can enter an individual’s problem representation as health-related cues; everyday activities therefore provided concrete experiential reference points for changes in hand function (23). Research on functional awareness in MCI provides further context for understanding differences among participants. A study comparing self-ratings with performance-based assessments across six domains of instrumental activities of daily living among individuals with amnestic MCI found that awareness accuracy varied across functional domains and MCI subtypes. Discrepancies between self-evaluation and actual performance were particularly evident in activities involving complex information processing and were not uniformly present across all domains (34). This evidence suggests that differences in awareness in the present study should be understood in relation to both individual variation in functional self-appraisal and the extent to which everyday life provides opportunities to use relevant hand abilities and compare current with previous performance. Participants’ uncertainty may therefore reflect an interplay between functional self-appraisal and activity experience, rather than a generalized state of awareness detached from specific contexts.

Everyday activities also provided a basis for judging the practical significance of change. When hand-related difficulties disrupted familiar and valued activities, the changes were experienced through reduced efficiency, restricted participation, or a diminished sense of competence. By contrast, when participants’ current routines placed few demands on the relevant hand abilities, recognized differences could still be perceived as having limited impact. This pattern is consistent with the International Classification of Functioning, Disability and Health, which conceptualizes functioning as an interaction among body functions, activities, participation, and contextual factors rather than as a direct reflection of impairment (35). This relationship is particularly relevant in the context of MCI. A systematic review of 37 studies found that overall independence may remain largely preserved in MCI, while subtle difficulties can already emerge in cognitively demanding instrumental activities of daily living, manifesting as slower completion, reduced accuracy, or lower efficiency rather than a complete loss of ability (6). Functional vulnerability at the MCI stage may therefore become visible when the demands of specific activities no longer align with an individual’s abilities, rather than presenting as a generalized and readily apparent loss of function. Everyday activities provide both the contexts in which such mismatches can be recognized and the practical basis on which participants judge whether changes have affected their independence, participation, and sense of self.

### 4.2 Hand-function change occupied an ambiguous interpretive space between aging and cognitive health

Participants primarily understood changes in hand function within the context of aging and age-related bodily decline, although their views about a possible relationship between hand function and cognitive function varied. Interpreting changes in hand function as part of aging was plausible, as normal aging can affect multiple components of hand function, including strength, sensation, dexterity, and coordination (10, 11). In everyday Chinese discourse, the expression “this is what happens when people get old” provides a familiar and readily available framework for making sense of bodily changes. Previous qualitative studies in China have similarly shown that aging may be understood both as a natural and largely uncontrollable process and in terms of continued engagement in life and active health maintenance (36, 37). These different meanings were also reflected in the present study. Some participants regarded changes in hand function as natural and acceptable limitations of later life; others emphasized the inevitability and limited modifiability of declining ability; and some, while acknowledging the role of aging, continued to express determination or a desire to try again. Older adults’ perceptions of aging have been associated with physical and cognitive functioning, activities of daily living, help-seeking, self-rated health, and quality of life (38). Thus, a shared attribution to aging could encompass different judgments about the normality, threat, and controllability of changes and was described alongside different evaluations of hand-function change and orientations toward action.

This interpretive ambiguity is particularly salient in the context of MCI. Research on illness representations in MCI has found that memory and language difficulties are more readily recognized as manifestations of MCI than bodily changes commonly experienced in later life (39). Performance-based studies, however, have identified selective differences between older adults with MCI and cognitively healthy peers in finger-tapping frequency, rhythm, performance variability, and complex manual dexterity, whereas simpler measures such as grip strength have not shown consistent differences (16–18). These findings suggest that some aspects of complex hand performance may have cognitive-motor relevance to MCI, although this relevance neither corresponds closely to everyday understandings of MCI centered on memory and language difficulties nor presents as a generalized and readily apparent loss of hand function. At the same time, these group-level cognitive-motor associations provide a context for understanding potential hand-function vulnerability in MCI; at the individual level, normal aging, other physical conditions, and MCI-related processes may offer overlapping explanations. The ambiguity therefore arises from the intersection of two plausible interpretations: aging provides a familiar and evidence-based explanation for changes in hand function, while MCI-related cognitive-motor vulnerability may be expressed in subtler and more task-dependent ways.

From a CSM perspective, causal attribution constitutes only one component of a health-related problem representation. Individuals also organize their understanding around identity—the label assigned to the change and its associated manifestations—timeline, consequences, and controllability, while illness coherence reflects the extent to which the health problem is understood as a coherent whole (23, 40). In the present study, attribution to aging provided an answer to why hand function might change, but participants varied in the extent to which they integrated this explanation with the nature of the change, the possible hand-cognition relationship, its everyday consequences, and its controllability. Some participants articulated one or more of these aspects clearly, whereas others remained uncertain. Changes in hand function therefore occupied an ambiguous interpretive space between aging and cognitive health: they had acquired an accessible causal explanation, but this explanation was not consistently integrated into a coherent understanding of their nature, functional significance, and controllability. These differences in problem representation were considered alongside participants’ descriptions of coping, particularly whether valued activities were linked to concrete strategies and available support.

### 4.3 Differences in coping centered on whether valued activity goals could be linked to feasible strategies and available support

In participants’ accounts, concrete coping responses were most often described together with a connection between an activity or ability they wished to maintain and a feasible course of action. Using a shopping trolley, modifying how tasks were performed, practicing finger exercises, and accepting assistance from others provided specific pathways for continuing to shop, maintaining hand flexibility, or completing difficult everyday tasks. By contrast, some participants noticed changes in hand function or valued particular hand abilities but had not considered specific training, were uncertain whether relevant activities would improve their condition, or described no clear coping response because the changes were perceived as having little impact on their current lives. From a CSM perspective, representations of the nature, causes, consequences, and controllability of a change provide reference points for coping; translating these representations into action also involves a practical understanding of what can be done and how it can be implemented in everyday life (23). Previous qualitative research among community-dwelling older adults with MCI has similarly identified limited perceived control and insufficient information and support as common barriers in accounts of self-management (41). Research involving structured guidance has shown that people with MCI can identify personally meaningful goals related to instrumental activities of daily living, leisure, and social participation (42). Goal-oriented interventions incorporating individualized strategies and the involvement of a support person have also been shown to improve goal performance and satisfaction among people with MCI or early-stage dementia (43). Together, this evidence provides a context for understanding differences in coping in the present study: valued activity goals may provide a starting point for action, while their implementation can be supported by connections with concrete strategies, assistive tools, and available support.

### 4.4 Coping after existing activities became constrained centered on goal maintenance and adjustment of engagement, with goal reengagement less prominent

Beyond the availability of concrete pathways for action, differences in coping were also evident in how participants maintained, adjusted, or reduced their engagement with an activity goal when the activity remained constrained by difficulties in hand function. Two modes of regulating goals under conditions of limited capacity have been distinguished: individuals may seek to maintain an existing goal by modifying their actions or external circumstances, or they may adjust the goal or their expectations in response to perceived constraints (44). From this perspective, the task modifications, assistive devices, and assistance from others described above differed in form but all altered the means through which an activity goal could be pursued, thereby allowing the original activity to continue. Reducing shopping or discontinuing knitting, by contrast, reflected decreased engagement with the original activity. Goal disengagement does not necessarily occur immediately after difficulties arise but may develop through repeated obstruction and frustration (45). The period during which individuals repeatedly weigh continued engagement against withdrawal from a goal has been conceptualized as an “action crisis” (46). This process-oriented perspective offers one way of interpreting how emotional experiences were described alongside different patterns of activity engagement in the present study. Wanting to try again and expressions of determination indicated continued commitment to an activity or ability; frustration and self-doubt reflected tension between continued effort and experienced difficulty; and calm acceptance or resignation accompanied reconsideration of personal capacity and activity demands. These emotional experiences therefore provided insight into whether participants were maintaining, reassessing, or gradually reducing their engagement with an existing activity. When participants situated the discontinuation of a difficult activity within the explanation that “this is what happens when people get old,” acceptance concerned not only the change in hand function itself but also a reduction in commitment to the activity.

A goal-adjustment framework extends the analysis from whether an existing activity was discontinued to whether the commitment and personal meaning associated with that activity were subsequently carried forward into another attainable goal (47). Within this framework, withdrawing effort and commitment from a blocked goal constitutes goal disengagement, whereas redirecting commitment toward another attainable and valued goal constitutes goal reengagement. Applied to the present findings, most concrete strategies served to maintain existing activity goals. Reducing shopping or discontinuing knitting reflected decreased engagement with those activities, whereas accounts of redirecting commitment toward alternative attainable and valued activities were less prominent. This pattern has particular relevance in the context of MCI. In a behavior-change intervention, older adults with MCI were able to set goals and make progress, but more often modified existing behaviors than established new goals; participants also reported that additional sessions or continued contact might help them progress toward and maintain their goals (48). Considered alongside previous goal-oriented research in MCI, coping in the present study primarily centered on maintaining familiar activities, modifying how they were performed, and reducing engagement when they became difficult. Developing a new and meaningful direction after a valued activity becomes constrained may represent a further self-regulatory task supported by clearer strategies and continuing assistance.

### 4.5 Implications for research and clinical practice

The clinical relevance of this study lies in showing that noticing, interpreting, and responding to subtle changes in hand function may each be present to different degrees among older adults with MCI. Clinical and community-based assessments should therefore attend to the activity contexts in which changes arise, the meanings attributed to them, and the availability of feasible pathways for action. Assessment could begin with familiar and personally meaningful activities by asking whether hand performance during writing, grasping objects, opening containers, shopping, or completing household tasks has changed from previous performance. When a change is reported, further inquiry could explore how the individual explains it, its practical consequences, its perceived controllability, and the activities or abilities the person wishes to maintain. Depending on the activity difficulty described, assessment could also incorporate relevant task performance and specific components of hand function, including strength, dexterity, movement speed, and coordination, to provide a fuller understanding of the relationship between subjective experience and observed performance. Support could be organized around personally valued activity goals by connecting these goals with task modifications, assistive devices, appropriate practice, and assistance available from others. For activities that have been reduced or discontinued, practitioners could explore whether they continue to carry important meanings related to leisure, participation, or competence and consider alternative ways of performing the activity or other activities through which similar meanings could be maintained. Goal-oriented intervention research suggests that older adults with MCI can learn and implement strategies related to personally meaningful goals, although some may benefit from additional explanation, opportunities for practice, ongoing contact, or the involvement of a support person to facilitate the understanding and maintenance of these strategies in everyday life (43, 48).

Future research could pursue two main directions. First, qualitative accounts and self-appraisals could be combined with informant reports and objective assessments of strength, dexterity, movement speed, and coordination, together with a cognitively healthy comparison group. Such research could examine the relationship between subjective experiences and observed hand performance and identify the activity contexts in which subtle cognitive-motor vulnerability associated with MCI may become most apparent. Second, longitudinal designs could examine how changes in hand function are noticed and reinterpreted over time and how activity goals, emotional experiences, perceived controllability, and available support accompany continued effort, task modification, activity reduction, and goal reengagement. Direct examination of activity participation, social support, and views of aging could further clarify the contextual basis of individual differences.

### 4.6 Strengths and limitations

This study examined how subtle changes in hand function were perceived, interpreted, and responded to in everyday life from the perspectives of community-dwelling older adults with MCI, adding qualitative evidence on subjective experience and functional meaning to previous research based predominantly on objective measurement. The CSM provided a systematic framework for the interviews and analysis while allowing openness to new meanings emerging from participants’ accounts. Purposive sampling included participants of different ages, genders, and educational backgrounds, while concurrent data collection and analysis, comparison of coding between researchers, team discussions, and maintenance of an audit trail further enhanced the trustworthiness of the study.

The findings should be interpreted in light of several study characteristics. Participants were recruited primarily from communities in Nanjing. Although the sample included older adults with diverse demographic backgrounds, its size did not support reliable subgroup comparisons, and the transferability of the findings to other cultural and healthcare contexts requires further examination. Previous exercise and hand-activity experience, social support, and relevant health conditions were primarily based on participants’ self-reports and were not verified through medical records or specific clinical assessments. Although individuals with hand dysfunction attributable to identifiable musculoskeletal conditions, hand trauma, or neurological disorders were excluded, other systemic factors that may affect muscle strength and activity performance, including sarcopenia, may have contributed to some participants’ experiences. In the absence of a cognitively healthy comparison group and objective measures of hand function, the study describes subjective experiences in the context of MCI but cannot distinguish the relative contributions of MCI, normal aging, and other physical conditions. Finally, the single interviews captured participants’ current understandings and responses; longitudinal research is needed to examine how these processes develop over time.

## 5 Conclusion

This study describes how community-dwelling older adults with MCI recognized and interpreted subtle changes in hand function in relation to everyday activities. Familiar activities provided reference points for comparing current with previous performance and enabled participants to judge the practical significance of change in relation to activity efficiency, participation, and their sense of self. Participants commonly explained changes in terms of aging, but this explanation did not necessarily encompass the possible hand-cognition relationship, functional consequences, or controllability, leaving some changes in an ambiguous interpretive space between aging and cognitive health. Differences in coping were most evident in whether valued activities could be connected with feasible strategies, assistive devices, and support from others; responses centered on maintaining or modifying existing activities and reducing engagement when activities became difficult. The CSM provided a useful framework for understanding how functional cues were incorporated into problem representations and considered in relation to coping. These findings support the use of specific everyday activities in clinical and community-based assessments and the provision of feasible support tailored to the activities that older adults with MCI wish to maintain and to their learning needs. Future research combining longitudinal interviews, objective measures of hand function, and cognitively healthy comparison groups could further clarify how these experiences and coping processes develop over time.

## Data Availability

The datasets generated and analyzed during the current study are not publicly available because they contain potentially identifiable qualitative interview data. Due to confidentiality requirements and ethical restrictions, the full interview transcripts cannot be shared. Relevant anonymized excerpts supporting the findings are included in the article. Further inquiries can be directed to Jingjing Yan, yan4222@njucm.edu.cn.
