# Supplementary material for: Perceiving, interpreting and responding to changes in hand function among older adults with mild cognitive impairment: a common-sense model-informed qualitative study
Source: Front Psychiatry. 2026 Sep 21;17:1873688. doi: 10.3389/fpsyt.2026.1873688 (PMC13620760; doi:10.3389/fpsyt.2026.1873688)
Supplement: Supplementary file 1 [file Supplementaryfile1.docx]

This file provides the completed Consolidated Criteria for Reporting Qualitative Research checklist for this study.

**COREQ (COnsolidated criteria for REporting Qualitative research) Checklist**

| **Topic** | **Item No.** | **Guide Questions/Description** | **Reported in Section** |
| --- | --- | --- | --- |
| **Domain 1: Research team and reﬂexivity** | | | |
| *Personal characteristics* | | | |
| Interviewer/facilitator | 1 | Which author/s conducted the interview or focus group? | Methods (2.4) |
| Credentials | 2 | What were the researcher’s credentials? E.g. PhD, MD | Methods (2.4) |
| Occupation | 3 | What was their occupation at the time of the study? | Methods (2.4) |
| Gender | 4 | Was the researcher male or female? | Methods (2.4) |
| Experience and training | 5 | What experience or training did the researcher have? | Methods (2.4) |
| *Relationship with*  *participants* | | | |
| Relationship established | 6 | Was a relationship established prior to study commencement? | Methods (2.4) |
| Participant knowledge of the interviewer | 7 | What did the participants know about the researcher? e.g. personal goals, reasons for doing the research | Methods (2.4) |
| Interviewer characteristics | 8 | What characteristics were reported about the inter viewer/facilitator? e.g. Bias, assumptions, reasons and interests in the research topic | Methods (2.4) |
| **Domain 2: Study design** | | | |
| *Theoretical framework* | | | |
| Methodological orientation and Theory | 9 | What methodological orientation was stated to underpin the study? e.g. grounded theory, discourse analysis, ethnography, phenomenology,  content analysis | Methods (2.1, 2.6) |
| *Participant selection* | | | |
| Sampling | 10 | How were participants selected? e.g. purposive, convenience, consecutive, snowball | Methods (2.2) |
| Method of approach | 11 | How were participants approached? e.g. face-to-face, telephone, mail, email | Methods (2.2) |
| Sample size | 12 | How many participants were in the study? | Methods (2.3); Results |
| Non-participation | 13 | How many people refused to participate or dropped out? Reasons? | Results |
| *Setting* | | | |
| Setting of data collection | 14 | Where was the data collected? e.g. home, clinic, workplace | Methods (2.5) |
| Presence of non- participants | 15 | Was anyone else present besides the participants and researchers? | Methods (2.5) |
| Description of sample | 16 | What are the important characteristics of the sample? e.g. demographic data, date | Methods (2.2); Results; Table 2 |
| *Data collection* | | | |
| Interview guide | 17 | Were questions, prompts, guides provided by the authors? Was it pilot tested? | Methods (2.5); Table 1 |
| Repeat interviews | 18 | Were repeat inter views carried out? If yes, how many? | Discussion (4.6) |
| Audio/visual recording | 19 | Did the research use audio or visual recording to collect the data? | Methods (2.5) |
| Field notes | 20 | Were ﬁeld notes made during and/or after the inter view or focus group? | Methods (2.4, 2.5) |
| Duration | 21 | What was the duration of the inter views or focus group? | Methods (2.5) |
| Data saturation | 22 | Was data saturation discussed? | Methods (2.3) |
| Transcripts returned | 23 | Were transcripts returned to participants for comment and/or | Not reported |

| **Topic** | **Item No.** | **Guide Questions/Description** | **Reported in Section** |
| --- | --- | --- | --- |
|  |  | correction? |  |
| **Domain 3: analysis and findings** | | | |
| *Data analysis* | | | |
| Number of data coders | 24 | How many data coders coded the data? | Methods (2.6) |
| Description of the coding tree | 25 | Did authors provide a description of the coding tree? | Methods (2.6); Table 3 |
| Derivation of themes | 26 | Were themes identiﬁed in advance or derived from the data? | Methods (2.6); Table 3 |
| Software | 27 | What software, if applicable, was used to manage the data? | Methods (2.6) |
| Participant checking | 28 | Did participants provide feedback on the ﬁndings? | Not reported |
| *Reporting* | | | |
| Quotations presented | 29 | Were participant quotations presented to illustrate the themes/ﬁndings? Was each quotation identiﬁed? e.g. participant number | Results (Themes 1–7) |
| Data and ﬁndings consistent | 30 | Was there consistency between the data presented and the ﬁndings? | Results; Table 3 |
| Clarity of major themes | 31 | Were major themes clearly presented in the ﬁndings? | Results; Table 3 |
| Clarity of minor themes | 32 | Is there a description of diverse cases or discussion of minor themes? | Results; Table 3 |

Developed from: Tong A, Sainsbury P, Craig J. Consolidated criteria for reporting qualitative research (COREQ): a 32-item checklist for interviews and focus groups. *International Journal for Quality in Health Care*. 2007. Volume 19, Number 6: pp. 349 – 357
