# Supplementary material for: Perceiving, interpreting and responding to changes in hand function among older adults with mild cognitive impairment: a common-sense model-informed qualitative study
Source: Front Psychiatry. 2026 Sep 21;17:1873688. doi: 10.3389/fpsyt.2026.1873688 (PMC13620760; doi:10.3389/fpsyt.2026.1873688)
Supplement: Supplementary file 2 [file Table1.docx]

**Supplementary Table 1.**

**Individual sociodemographic and clinical characteristics of participants (N = 19)**

| Participant ID | MoCA score | Age  (years) | Gender | Years of education | Marital status | Living arrangement | Monthly disposable  income (CNY/month) | Pre-retirement occupational  work type |
| --- | --- | --- | --- | --- | --- | --- | --- | --- |
| N1 | 23 | 75 | Female | 12 | Married | Living with children | 3,001-4,000 | Mental work |
| N2 | 24 | 68 | Female | 15 | Married | Living with spouse and children | 1,000-2,000 | Mixed mental and manual work |
| N3 | 22 | 73 | Female | 12 | Widowed | Living alone | 2,001-3,000 | Manual work |
| N4 | 22 | 82 | Female | 16 | Widowed | Living alone | ＞5,000 | Mental work |
| N5 | 24 | 72 | Male | 9 | Married | Living with children | 1,000-2,000 | Mental work |
| N6 | 20 | 70 | Male | 9 | Married | Living with spouse | < 1,000 | Manual work |
| N7 | 23 | 68 | Male | 9 | Married | Living with spouse and children | 1,000-2,000 | Manual work |
| N8 | 23 | 71 | Female | 15 | Married | Living with spouse and children | 3,001-4,000 | Mental work |
| N9 | 25 | 72 | Female | 9 | Married | Living with spouse | 4,001-5,000 | Mixed mental and manual work |
| N10 | 20 | 70 | Male | 9 | Married | Living with children | 2,001-3,000 | Mental work |
| N11 | 25 | 82 | Male | 15 | Married | Living with spouse | ＞5,000 | Mixed mental and manual work |
| N12 | 23 | 75 | Female | 12 | Married | Living with spouse | ＞5,000 | Mixed mental and manual work |
| N13 | 25 | 79 | Male | 15 | Married | Living with spouse | 3,001-4,000 | Mental work |
| N14 | 23 | 68 | Female | 12 | Married | Living with spouse | 1,000-2,000 | Manual work |
| N15 | 24 | 71 | Male | 12 | Married | Living with spouse | 1,000-2,000 | Mental work |
| N16 | 18 | 77 | Female | 6 | Widowed | Living alone | 3,001-4,000 | Mixed mental and manual work |
| N17 | 25 | 72 | Female | 12 | Widowed | Living alone | ＞5,000 | Mental work |
| N18 | 24 | 72 | Female | 15 | Married | Living with spouse | ＞5,000 | Mental work |
| N19 | 24 | 76 | Female | 15 | Widowed | Living alone | ＞5,000 | Mental work |

*Note.* MoCA, Montreal Cognitive Assessment; CNY, Chinese yuan. Monthly disposable income was self-reported and recorded using predefined categories. Pre-retirement occupational work type was classified according to the main demands of participants’ previous occupations: mental work refers to occupations primarily involving administrative, professional, clerical, or cognitively oriented tasks; manual work refers to occupations primarily involving physical labor; and mixed mental and manual work refers to occupations involving both cognitive and physical demands. Participant IDs are anonymized study codes.
